# Social Construction and Evolutionary Perspectives on Gender Differences in Post-traumatic Distress: The Case of Status Loss Events

**DOI:** 10.3389/fpsyt.2022.858304

**Published:** 2022-05-16

**Authors:** Roy Azoulay, Eva Gilboa-Schechtman

**Affiliations:** Department of Psychology and Gonda Multidisciplinary Brain Center, Bar-Ilan University, Ramat Gan, Israel

**Keywords:** trauma, gender, social-rank, evolutionary psychology, humiliation, status, sex, PTSD

## Abstract

Women report greater post-traumatic distress (PTD) than men following physically threatening events. However, gender differences in PTD following social stressors such as status losses are understudied. Whereas the social construction account points to a general sensitivity in women following any type of stressor, the evolutionary account suggests enhanced sensitivity to status losses in men, especially following inter-males aggressions. These propositions were examined in two studies (Study 1, *N* = 211; Study 2, *N* = 436). Participants were asked to recall a status loss and to fill out measures assessing PTD and depression severity. In line with the evolutionary account, men, as compared to women, displayed enhanced PTD following status loss. Status losses conducted by men against men were associated with greater PTD than were instances involving other target-aggressor pairings. Finally, age was negatively associated with PTD in men but not in women. The examination of evolutionary challenges modifies the standard view linking the female gender to enhanced sensitivity to trauma. Thus, the pattern of enhanced sensitivity to stressful events appears to be affected by gender- and development-specific adaptive challenges.

## Introduction

Studies have shown small to moderate effect sizes of gender on the prevalence of post-traumatic distress (PTD) following exposure to trauma [For review: (1)]. Women are diagnosed with post-traumatic stress disorder approximately twice as often as men (2), and report higher levels of both re-experiencing, avoidance, and arousal symptoms (3). Furthermore, consistent meta-analysis results documented enhanced PTD in women compared to men following a wide variety of events including assaults, accidents, disasters, combat of war and injury or death witnessing, whereases enhanced PTD in men compared to women was not found following any type of stressor (4).

However, the scope of these gender differences is debated. On the one hand, the social construction account points to a *general* sensitivity in women due to lower (perceived and actual) social status and a propensity for more internalized coping styles (5). The evolutionary account, in contrast, suggests that the traumatic impact of an event is associated with its *interruption* of sociobiological goals, and thus, is likely to differ between the genders (6, 7). Specifically, evolutionary theorists argue that whereas women tend to be more susceptible to physical threats, men are more sensitive to status losses (8–10). Notably, most studies examining gender differences and PTD focused on physically threatening events [falling inside Criterion A definition in the DSM-V, (11, 12)]. Clearly, such data do not differentiate between the predictions of the social construction and the evolutionary accounts. Examining gender differences in response to status loss events (SLEs; e.g., humiliation, demotion) which are known to provoke PTD (13), is central to differentiating between the two theories. This is the main goal of the present study.

### Social Construction Theory

According to social construction theorists, women’s propensity to develop PTD is explained by their perceived lower status, feelings of powerlessness, and reinforced shame responses following traumatic events (5). According to this reasoning, PTD is expected to be more severe in women following *any type* of traumatic event (including SLE). Indeed, low-status individuals report higher distress following SLEs (14), and feelings of powerlessness or increased activation of shame responses contribute to reported distress following such events (15–17). Notably, if women’s enhanced PTD originates from status deficiency as suggested by social constructionists, that tendency is expected to be present, and even emphasized, in events that compromise social status.

Furthermore, the social construction account suggests that, due to status gender inequality, women tend to encounter more SLEs instigated by men (18, 19). Importantly, according to this account, because traditional male gender roles emphasize dominance and power, men are more prone to challenge the status of women, often as an inter-gender aggressive means to maintain low status in women and enhance men’s own social standing (5, 18, 19). Notably, women experience more shame and display more submissive behaviors following SLEs that are committed by men (20, 21). Thus, according to the social construction theory, SLEs conducted by men against women are suggested to be more traumatic than other types of victim-aggressor gender combinations.

Finally, according to the social construction theory, gender-roles are predicted to become more salient with age due to continuous endorsements of social constructs (22). Because gender-roles predict response to trauma more than biological gender does, PTD severity in women is expected to *increase* with age (23). Moreover, the status differential between genders increases throughout adulthood (24, 25), again consistent with women’s predicted vulnerability to SLEs. In summary, social construction theory is consistent with a *generalized* scope of women’s vulnerability and predicts that such vulnerability echoes the women’s endorsement of their traditional cultural gender-roles.

### Evolutionary Theory

According to the evolutionary perspective, SLEs reduce access to resources and mating options for both genders (26, 27, 94, 96). However, status is more strongly linked to reproductive prospects and wellbeing among men, than among women (28–30). Importantly, status change in men is often determined by *single* events (such as SLE) whereas in women this change tends to be associated with continuous accumulation of events (31, 32). Moreover, SLEs were found to affect objective markers of social dominance (e.g., testosterone) to a greater extent in men than in women (33, 34). Taken together, evolutionary theories suggest that, as compared to men, men are likely to be more susceptible to severe PTD following SLEs.

Evolutionary models further suggest that status is attained differently among men and women (35), and is more preferentially determined *via* intrasexual conflicts among men [(36); *p. 429*; (37, 98)]. Indeed, replicated findings indicate that social status in males, but not females, is strongly associated with the (perceived and actual) ability to physically win intrasexual conflicts [for reviews, see (37, 38)]. Notably, the exercise of status-related physical inter-males competition was partially replaced by knowledge- and skills-based competition among humans (39). Moreover, in primates, losing in intrasexual conflicts is the most common precursor to social demotion only among males (32, 40, 41). Accordingly, evolutionary accounts indicate that sensitivity for SLEs may be enhanced when both the aggressor and the victim of SLEs are men.

Finally, evolutionary theorists expect status concerns to mirror men’s fertility (42). Consequently, status concerns are predicted to be weakened by age due to age-related reductions in reproductive goals (43, 44). Indeed, testosterone levels decline with age especially among men (45). Moreover, results based on large samples document that discrepancy in status motivations between men and women, which emerges in adolescence and persists throughout early adulthood, is diminished in late adulthood (46). Hence, the enhanced sensitivity in men in response to SLEs is suggested to be age dependent.

Taken together, evolutionary models highlight the differences between women and men’s reproduction strategies which map onto discrepancies in psychological features such as anxiety, intra-gender aggression and status seeking (47, 48, 99). Those discrepancies are postulated to be amplified in early adulthood, when reproduction goals are most salient (49). Accordingly, the predictions of the evolutionary theory are consistent with a limited and specific scope of vulnerability in women which mirrors the activation of survival and reproductive goals.

## Current Research

The aim of the current research is to contrast the social construction and the evolutionary theories regarding gender differences in PTD following SLEs. Specifically, according to the social construction account, SLE would induce more severe PTD in women as compared to men, especially when the aggressor is a man. Furthermore, because the status differential between genders increases with age (24), women’s sensitivity to SLE is expected to increase with age. In contrast, the evolutionary theory hypothesizes that SLEs would induce more severe PTD in men compared to women, especially following SLEs that were carried out by other men. Finally, the evolutionary account further suggests that the enhanced PTD among men following SLE would decrease with age.

Accordingly, we contrasted three pairs of hypotheses. First, we hypothesized that women would differ in their PTD levels compared to men following SLEs (the *men sensitivity vs. women sensitivity hypotheses*); Second, we hypothesized that the gender of the aggressor would affect PTD levels. Specifically, we predicted based on evolutionary theories that SLEs conducted by men against men would be most traumatic (the *inter*-*males aggression hypothesis)* or that in accordance with social construction theories, SLEs conducted by men against women would be the most distressing (the *males against females aggression hypothesis)*. Finally, we expected that gender and age would interact to predict PTD. In line with the evolutionary account, we predicted that SLEs would correlate with age especially among men (the *age-men link hypothesis)*, whereases based on the social construction theory we predicted that age would correlate especially with PTD among women (the *age-women link hypothesis)*.

Two studies were conducted to address these hypotheses. In both studies, we asked participants to recall an SLE and report on event related, as well as general, measures of distress. In the first study we invited participants who encountered a *significant* SLE (*N* = 212), whereas in the second study we included all individuals who were able to identify *any* specific SLE experience (*N* = 436). Notably, because detecting interaction in regression requires a sample size four times larger than that requires to detect the main regression effect (50), we examined the age-related PTD hypotheses by combining our two samples. Furthermore, depression was included as a covariate due to its robust association with distress following SLEs (51).

## Method

### Study 1

#### Participants

Based on the reported moderate effects in studies that investigated the relation between gender and PTD (4), a sample size of 210 was chosen as providing sufficient power for identifying the anticipated effects [G*Power 3.1; (52)]. A greater number of participants (*N* = 374) was recruited based on the exclusion rate in prior similar studies (53). Participants were recruited *via* the Amazon Mechanical Turk (MTurk) platform and received 5$ for their participation. All participants were from the United States with English as their native language. Exclusion criteria were: (a) filling out the survey from an I.P used by another participant/s [(53); *n* = 37]; (b) completing the autobiographical task in a non-conscientious manner (i.e., writing irrelevant text in the description of the memory as assessed by the two authors; *n* = 126). The final sample consisted of 211 participants (80 women). Participants’ ages were between 22 and 69 (Mean = 36.7; SD = 10.2). The average number of education years was 15.1 (SD = 4.1).

#### Procedure

Participants were invited to take part in a 30-min survey geared to understand responses to severely stressful social events. After filling a consent form, participants were requested to recall an SLE. Following the recall, the participants were asked to indicate the age at which the recalled event occurred and the gender of their aggressor(s) (a man, a woman, or both). Next, they filled out PTD and depression severity questionnaires. Finally, they completed a series of demographic questions (e.g., age, education, gender) and were thanked and debriefed. All measures were administered in English.

#### Measures

*Recollection of status loss* was induced by asking participants to recall an event in which they “*felt belittled or that their dignity was compromised by others.”* Next, they were asked to write a detailed description (at least 50 words) of the event. The instructions were based on the recall task used by Tangney et al. (54), which is designed to examine the characteristics of unpleasant social memories. Importantly, to modify the task for recollection of SLEs, we used Klein (55) definition for humiliation (an event in which one is being belittled or treated with indignity). In order to examine whether participants recalled SLEs, the two authors read all narrative independently. Narratives that were not social or did not include a threat to status were excluded (Inter-rater reliability = 0.96). Furthermore, to evaluate whether the recall task induced memories which are perceived as loss of status, we asked participants to rate their emotions during the event on five emotions scales (Humiliation, Shame, Sadness, Guilt, Anxiety). As expected, emotions which are associated with status loss such as humiliation and shame were significantly higher compared to the other negative emotions [*F*(209,1) = 186.60, η*_*p*_^2^* = 0.33].

*Post-traumatic distress (PTD)* was assessed using the Post-traumatic Diagnostic Scale for DSM-V [PDS-5; (56)]. In PDS-5, the symptom items are rated on a scale of frequency and severity. Specifically, the scale includes items assessing intrusion (e.g., *Unwanted upsetting memories about the event*), avoidance (e.g., *Trying to avoid thoughts or feelings related to the even*t), negative cognitions and mood (e.g., *Having intense negative feelings like fear, horror, anger, guilt or shame*), and arousal (e.g., *Being jumpy or more easily startled*). Because the sample included participants who did not perceive the event as a trauma, the word “trauma” in the questionnaire was replaced with the word “event.” The use of post-traumatic measures to assess PTD following socially stressful events was found to be reliable (13) and was constantly applied to assess PTD following SLEs (13, 57–59). The internal reliability of the scale was 0.85.

*Depression severity* was assessed using the Beck Depression Inventory [BDI–II; (60)], consisting of 20 items (the suicidality item was excluded due to the online nature of the study). The internal reliability of the questionnaire in our sample was 0.91.

### Results

Descriptive statistics are presented in Table 1. As can be seen in the Table, there were no significant differences between men and women in age, trauma recency, or depression. To examine the *gender-sensitivity hypotheses*, we first conducted an ANCOVA with PTD as a dependent variable, gender as an independent variable, and depression and age as covariates. Consistent with the evolutionary account, we found a main effect for gender [*F*(1,207) = 5.68, *p* = 0.01, η*_*p*_^2^* = 0.03], such as that the PTD-severity was greater for men than for women (Table 1). Next, we examined the percentage of participants whose PDS scores were above the cutoff for PTSD [PDS Score above 28; (56)]. Again, we found a significant main effect of gender [*X*^2^(1) = 5.91, *p* = 0.01]. Notably, men were more likely than women to meet the PTSD-cutoff.

**TABLE 1:** Age, depression, and event related factors among men and women.

	Study 1		Study 2	
	Women (*N* = 80)	Men (*N* = 131)	Women (*N* = 250)	Men (*N* = 187)
Age	39.3 (11.4)	35.1 (9.1)	42 (13.1)	38.9 (12.5)
Trauma Recency	13.2 (12)	10.6 (9.4)	12.1 (14.3)	11.0 (10.9)
Depression Severity	17.4 (14.8)	19 (14.8)	10.6 (11.9)	11.4 (10.9)
Post-traumatic distress	24.4 (23.8)^A^	32.6 (24.7)^B^	12.7 (17)^A^	18 (11.1)^B^
% PTSD	***40*** ^**A**^	***57*** ^**B**^	***7*** ^**A**^	***18*** ^**B**^
% Men as aggressors	***47***	***77***	***42***	***58***

*Mean; (Standard deviation); Means at the same row and study that do not share the same superscript differ at p < 0.05.*

**FIGURE 1:**
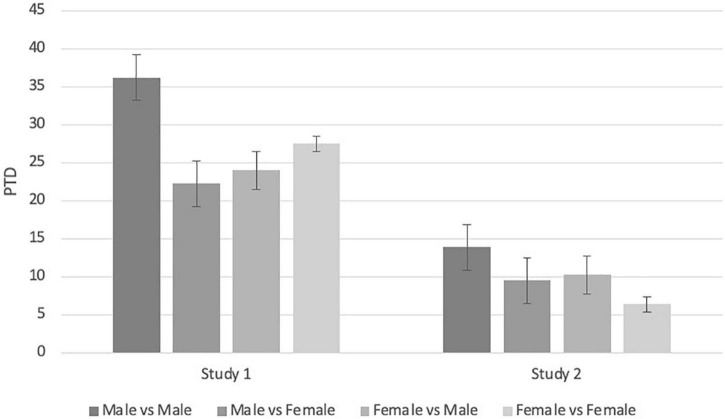
Post-traumatic distress severity by gender of victim and aggressor. PTD, post-traumatic distress as measured by PDS-V.

Next, we examined the *aggression* hypotheses comparing events in which both the aggressor and the victims were men (*N* = 101) and events in which the aggressor was a man and the victim was a woman (*N* = 35). In line with the evolutionary account, SLEs that were conducted by men against men were associated with a more severe PTD compared to SLEs in which the aggressors were men and the victim woman [*t*(187) = 2.02, *p* = 0.008, Cohen’s *d* = 0.58; Figure 1]. We further conducted a contrast between events in which both the aggressor and the victim were men and the three other aggressor-victim configurations (i.e., woman-man, man-woman, and woman-woman). In line with the evolutionary account, the contrast was significant [*t*(187) = 4.64, *p* < 0.001, Cohen’s *d* = 0.67].

To sum, both hypotheses of the evolutionary account were supported. However, because we invited participants that define their recalled event as impactful, it is possible that gender differences are explained by higher prevalence or accessibility of impactful SLEs among men, as compared to women. To address this possibility, in Study 2 we invited participants who could recall a specific SLE regardless of the level of its impact.

### Study 2

#### Participants

Based on the small effect size found in Study 1, and because we anticipated an even smaller effect due to the inclusion of less intense social events, a sample size of 400 was chosen to provide sufficient power for identifying the anticipated effects [G*Power 3.1; (52)]. Based on the exclusion rate in similar prior studies, 455 participants were recruited *via* TurkPrime, which enables to recruit more conscientious Mturk workers (61). All participants were from the United States with English as their native language. Exclusion criteria were: (a) filling out the survey from an I.P used by another participant/s (*n* = 9); (b) completing the recall task in a non-conscientious manner (i.e., writing irrelevant text in the description of the memory as assessed by the two authors; *n* = 10). The final sample consisted of 436 participants (250 women). Participants’ ages were between 18 and 79 (Mean = 40.6; SD = 13.0). The average number of education years was 15.5 (SD = 2.3).

#### Procedure

The procedure was identical to Study 1 with one exception; Participants were invited to take part in a 30-min survey that sought to enhance our understanding of *unpleasant* social memories. All reliabilities of the scales were satisfactory as in Study 1 (PDS-5 = 0.87; BDI = 0.92).

### Results

As can be seen from Table 1, there were no significant differences between the genders in age, trauma recency, or depression. To test the first two hypotheses, we repeated the analyses from Study 1. As in Study 1, we found a main effect for gender in ANCOVA [*F*(1,433) = 15.96, *p* < 0.001,η*_*p*_^2^* = 0.04] and in Chi-square test [*X*^2^(1) = 14.2, *p* < 0.001] such as that the PTD score and estimated PTSD-diagnoses percentages were higher for men than for women (Table 1). We also found that inter-males SLEs were associated with more severe PTD compared to PTD following SLEs conducted by men against women [*t*(332) = 2.38, *p* = 0.02, Cohen’s *D* = 0.12; Figure 1]. As in Study 1, we contrasted events in which both the aggressor and the victims were men and the three other configurations: we found that inter-males SLEs were higher than the three other victim-aggressor type events [*t*(332) = 3.28, *p* < 0.001, Cohen’s *D* = 0.12].

**FIGURE 2:**
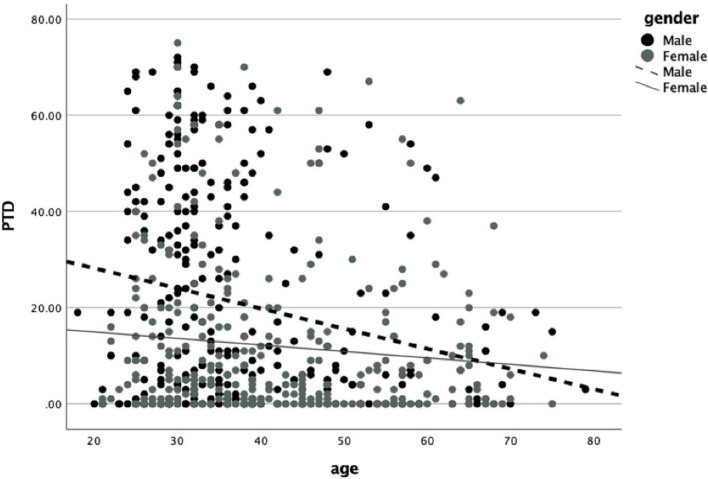
Linear regression for PTD predicted by age in Women (gray solid line) and Men (black dashed line). PTD, post-traumatic distress as measured by PDS-V.

Finally, combining the data from both samples, we examined *the gender-age link hypotheses.* A GLM was conducted with PTD as a dependent variable, and depression, age, gender (man = 1; woman = 2), and Gender × Age as predictors. A significant Gender × Age [β = 0.45, *b* = 0.31, *SE* = 0.1, *t*(646) = 2.95, *p* < 0.01; 95% CI(0.10,0.51)] interaction was found. Further analysis revealed that the age was associated with PTD severity in men but not in women (β = −0.11, *b* = −0.22, *SE* = 0.07, *p* = 0.01;β = −0.02, *b* = *0*.64, *SE* = 0.07, *p* > *0.5*, for men and women respectively; Figure 2).

## Discussion

The present study examined gender differences in PTD following status loss using two competing theoretical perspectives: social construction and evolution. Accordingly, three pairs of hypotheses were tested. First, we hypothesized that women would differ in their distress levels compared to men; Second, we hypothesized that the gender of the aggressor would affect distress levels. Finally, we expected that *gender and age would interact to predict* distress following status loss. Overall, our results are consistent with the evolutionary account. Specifically, women reported less severe PTD following status losses as compared to men, thus exhibiting greater resilience to these events. In addition, men reported severe PTD following events in which another man was a perpetrator. Finally, the PTD severity was unrelated to age in women, whereas in men this association was negative.

Our results joint those of van den Berg et al. (12) who reported gender differences in PTD following physical- but not social- stressors. Taking an evolutionary perspective, social and physical threats affect women’s and men’s reproductive success asymmetrically. Status is associated with low fertility and high offspring mortality among men but not women (62, 63), whereases women’s, but not men’s, fertility and attractiveness are highly linked with health (42). Differences in reproductive meanings of various stressors may partially account for gender discrepancy in PTD (6). For example, the cost of bodily harm may be relatively greater for women because of their central role in ensuring infant survival (64–66). Furthermore, among women, offspring survival is linked with a strong dyadic support network (67, 95), indicating that inclusionary events may be more influential for women, as compared to men.

We also found that the gender of the aggressor was associated with PTD severity. The genderial context in which a stressful event took place may affect the levels of elicited distress by threatening specific salient goals. For example, due to females’ high selectiveness, mating options for males are reduced and intra-sexual competition is enhanced (68) – leading to increased male sensitivity for intrasexual aggression (69). However, intra-sexual competition among females may take a different manifestation, such as covert verbal aggression (66, 70) or exclusion (71). Furthermore, the focus of the intrasexual competition may differ across genders. Whereas males tend more to compete on status, power, strength, and resources (47, 72), females’ competition resolve more around attractiveness and promiscuity (73–76). Future studies could examine what types of intrasexual aggression are most distressing in women.

Finally, for men, but not for women, age was found to be associated with ameliorated distress following status losses. Those results are in-line with evolutionary accounts emphasizing the enhanced prevalence of intrasexual aggressions among young males [“The young male syndrome”; (77)]. Furthermore, our age-gender interaction mirrors other gender discrepancies which declined with age and are status related such as risk-taking (78). Notably, the decline in status loss distress among men may reflects a decline in competitiveness due to the decrease of women’s reproductive value with age (79, 80). Importantly, the age-effect echoes gender-related differences in the prevalence of PTSD following physically threatening events which are reduced throughout adulthood (3, 81). It is possible that such changes in prevalence and severity mirror fluctuations in the levels of gonadal hormones associated with status motivations (34, 82). Specifically, reductions in testosterone levels in men and estradiol levels in women may contribute to the reduced gender discrepancy in the severity of distress following status losses (45).

## Theoretical Implications

The current findings add to the growing body of research demonstrating pervasive and deleterious post-traumatic effects of status losses(91, 92, 93). These findings support the claim that reproduction threats may engender full-blown PTSD, given that reproductive goals are comparable to, and sometimes even outweigh, survival goals (83, 97). From an evolutionary perspective, any life event that interferes with the achievement of short-term biological goals such as status can qualify as a trauma due to its relevance to biological adaptation in the ancestral environment (6). A focus on physical threats as sole potential PTSD-provokers narrows essential goals pursuit to the physical arena and dismiss the evolutionary importance of our social environment. Importantly, our results challenge the *general* women sensitivity hypothesis. Following a variety of events, women report higher distress compared to men (84). Ignoring the evolutionary context may over-emphasize, and even over-pathologize, women’s adaptive responses. As suggested by Troisi (6), social distress is induced following experiences which jeopardize sociobiological goals, as an adaptive response that facilitates the maintenance of the threaten goals. Thus, the distress is likely to be associated with the importance of sociobiological goals which is moderated by factors such as age and gender. Evolutionary theorists claim that to a certain level, gender differences in distress symptoms would remain, and that the gender gap in PTD could be narrowed by adopting a gender-sensitive nosology. Our results are in line with the latter position, indicating that gender by itself does not predict PTD and that its interaction with type of stressor need to be considered in PTSD classification.

## Limitations

In closing, several limitations of the present research need to be noted. First, our study relies on self-report measures which may lead to biased report of PTD and depression severity (85). Second, gender differences in PTD may reflect some yet untapped distinctions in nature of the recalled events. Future studies may rely on response to pre-scripted status losses scenarios and examine gender differences in anticipated distress. Third, evolutionary approaches suggest that status can be reduced *via* loss of dominance (experiencing physical or psychological intimidation) as well as prestige [incompetence to display valued skills and abilities; (86)]. The current study did not distinguish between losses of status *via* prestige from those losses *via* dominance. Whereases loss of prestige is predicted to affect both genders, loss of dominance is predicted to affect mostly men (87). Forth, we decided to use the gender terminology (and not the sex terminology) due to the self-report nature of our study. Specifically, participants were asked to indicate their gender and not their assigned sex at birth, thus only their gender identity was examined. Future studies could investigate whether the reported discrepancies are present when biological markers of sex are examined. Finally, our data does not distinguish whether men’s reactions to SLEs are less intense because it mirrors their fertility or because men tend to become more established in older age (and thus are less susceptible to status loss). Although prior studies indicate that both men and women tend to be more established with age, it is possible that the age-status enhancement affects more men than women (88).

## Conclusion

The last decades are witnessing an evolutionary turn in clinical psychology (89, 90). Psychopathologies are examined through adaptiveness framework and therapy is formulated as a mean to acquire flexible ways to navigate, toward and between, evolutionary goals. Women and men differ in their evolutionary challenges, and consequently in the type of events most relevant to those challenges. To date, studies documenting greater vulnerability of women to traumatic events did not consider variability in evolutionary-relevant goals and challenges. The evolutionary revolution is not complete without taking gender, age, and other survival and reproduction relevant variables into consideration. Consideration of these variables may help us appreciate the way the nature and timing of events individuals encounter on their unique journeys impact their development, identity, and wellbeing.

## Data Availability Statement

The original contributions presented in the study are included in the article/Supplementary Material, further inquiries can be directed to the corresponding author.

## Ethics Statement

The studies involving human participants were reviewed and approved by the Bar-Ilan University, Psychology Department. The patients/participants provided their written informed consent to participate in this study.

## Author Contributions

RA: conceptualization, methodology, software, formal analysis, investigation, data curation, and writing – original draft. EG-S: conceptualization, supervision, writing – review and editing, and funding acquisition. Both authors contributed to the article and approved the submitted version.

## Conflict of Interest

The authors declare that the research was conducted in the absence of any commercial or financial relationships that could be construed as a potential conflict of interest.

## Publisher’s Note

All claims expressed in this article are solely those of the authors and do not necessarily represent those of their affiliated organizations, or those of the publisher, the editors and the reviewers. Any product that may be evaluated in this article, or claim that may be made by its manufacturer, is not guaranteed or endorsed by the publisher.
